# A Single-organoid Workflow for quantitative Imaging classiFication and Tracking (SWIFT)

**DOI:** 10.1038/s42003-026-10768-x

**Published:** 2026-09-01

**Authors:** Lucie Bracq, Romain Guiet, Sandra Offner, Béatrice Kunz, Gisou van der Goot, Nathalie Brandenberg

**Affiliations:** 1 Global Health Institute, School of Life Sciences, EPFL, Lausanne, Switzerland; 2 BioImaging and Optics Core Facility, School of Life Science, EPFL, Lausanne, Switzerland

**Keywords:** Image processing, Intestinal stem cells

## Abstract

SWIFT (Single-organoid Workflow for quantitative Imaging classiFication and Tracking) is a fast and modular pipeline for quantitative, time-resolved organoid analysis from brightfield images. Requiring only minimal training annotations, SWIFT combines YOLOv8, SAM, and application-specific object classification to deliver accurate single-organoid segmentation, phenotyping, and tracking. The workflow is robust across organoid types and microscope systems. Applied to intestinal organoids, SWIFT uncovers dynamic Wnt-dependent morphological transitions, enabling scalable, high-throughput studies of epithelial plasticity.

## Abstract (web summary)

SWIFT is a fast and modular deep learning-based workflow for quantitative organoid analysis of brightfield images, enabling accurate single-organoid segmentation, phenotyping, and tracking with minimal training annotations.

## Introduction

Organoids have become indispensable models in biology, physiopathology and medicine as they recapitulate key features of stem cell niches, lineage differentiation, and certain organ functions in vitro^1^. They often surpass other experimental models, ranging from classical cell culture or animals, combining fidelity and scalability, but remain notoriously difficult to analyze due to their heterogeneity and dynamic behavior^2^. Intestinal organoids were the first to be developed and have continuously been refined now faithfully representing many intestinal processes in vitro^3,4^. They indeed capture phenotypic transitions that mirror in vivo cellular states, governed by tightly regulated signaling pathways such as Wnt^5^. Quantitatively analyzing this in vitro intestinal plasticity remains a bottleneck. Here we present a fast, versatile, and scalable image-analysis pipeline that enables precise tracking of organoid phenotypic changes in space and in time.

Brightfield imaging offers a non-invasive means to monitor organoids over time with high spatial and temporal resolution. Deep learning-based analysis pipelines have achieved high accuracy in organoid segmentation and classification, but typically require tens of thousands of manual annotations for training^6,7^. More recent studies used foundation models in computer vision, such as YOLO, which demand fewer annotations for fine-tuning and run faster, yet primarily rely on bounding-box detection, limiting their ability to perform precise segmentation and phenotypic classification^8^. Moreover, most published models are suitable for only a single type of organoid, and focus on static detection rather than tracing phenotype transitions over time^6–8^.

## Results and discussion

### Development and benchmarking of a deep-learning-based single-organoid detection-segmentation algorithm on brightfield images

**Fig. 1:**
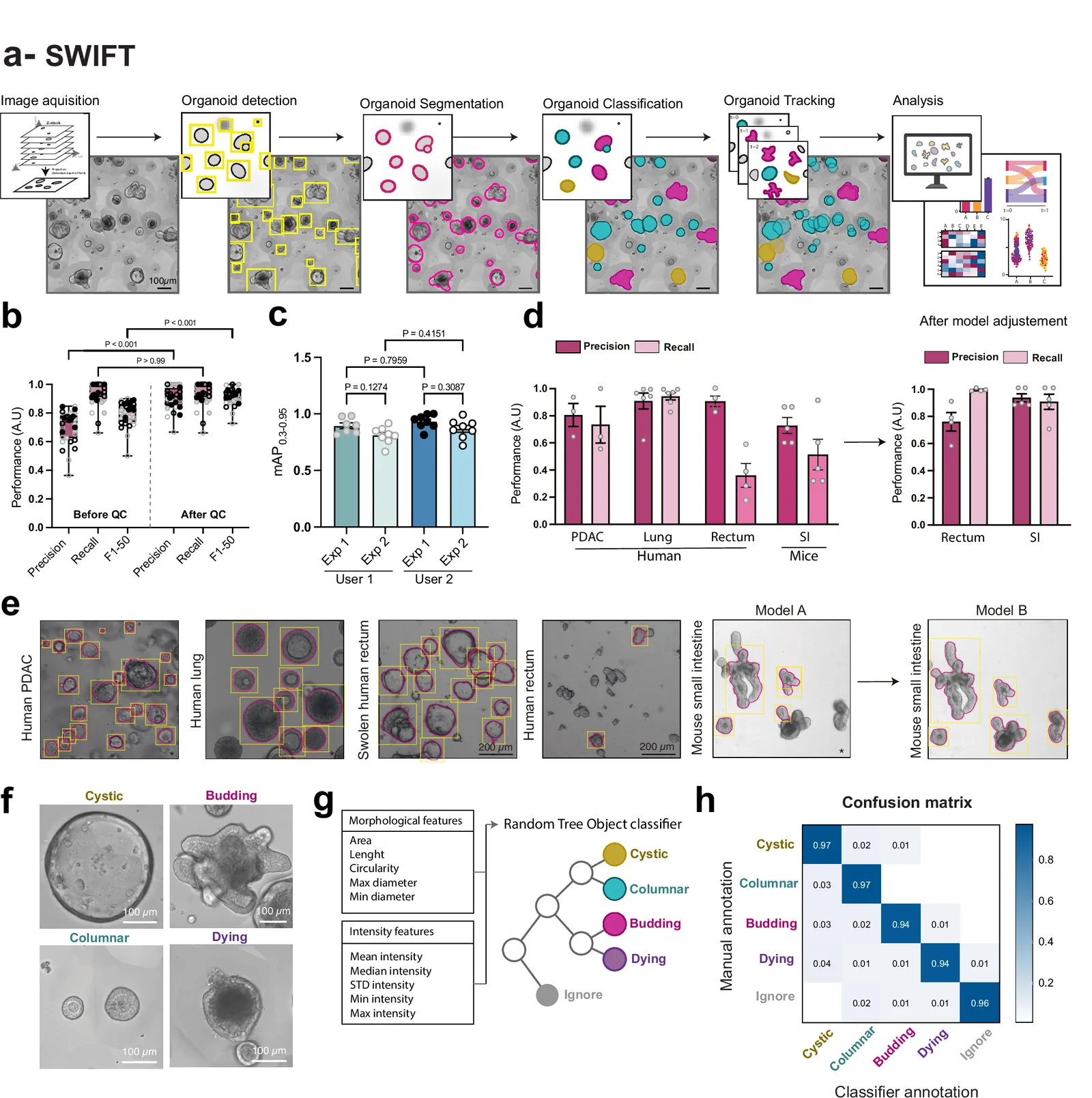
SWIFT: modular pipeline for single-organoid detection, segmentation, classification and tracking. **a** Overview of SWIFT workflow integrating organoid detection, segmentation, and classification for automated single-organoid analysis in time-lapse brightfield images. Scale bar: 100 µm. **b** Detection performance (mean ± SEM) of model A before and after quality control. Each dot represents the projection of one organoid well (96-well-plate). Black Vs gray dots represent two different users. Full versus empty dots represent two independent experiments. *n* = 8 wells per user and per experiment were quantified for a total of *n* = 32 wells. Two-way ANOVA with Sidak correction was performed. **c** Mean precision (mean ± SEM) across thresholds (mAP_0.3-0.95_) of model A across different experiments and users. Each dot represents the projection of one organoid well (96-well-plate). Black Vs gray dots represent different users. Full Vs empty dots represent two independent experiments. At least *n* = 7 wells per user and per experiment were quantified. Two-way ANOVA with Sidak correction was performed. **d** Performance (mean ± SEM) of model A (left panel), and B or C (right panel) on independent datasets. Each dot represents an image of publicly available datasets^7,21^. **e** Organoid detection (Yellow) and segmentation (magenta) with model A on PDAC, lung and with model B and C, respectively, on mouse small-intestine or human-rectum organoids from available datasets^7–21^. At least *n* = 3 images were quantified. Scale bar = 200 µm. * Scale bar non available from public datasets. **f** Brightfield images of cystic, columnar, budding, and dying organoids. Scale bar = 100 µm. **g** Schematics of morphometric and intensity features-based Random Tree object classifier in Qupath for phenotype classification. **h** Confusion matrix of classifier predictions versus manual annotations across morphological classes.

To address these limitations, we developed SWIFT (Single-organoid Workflow for quantitative Imaging classiFication and Tracking), a robust and modular pipeline for automated time-resolved analysis of organoid images. SWIFT integrates the deep learning-based object detection model YOLO (You Only Look Once)^9^. The segmentation model SAM (Segment Anything Model)^10,11^, and Random Tree object classifier implemented in QuPath^12^. Detected objects are first delineated by YOLO, automatically segmented by SAM, and subsequently classified and tracked in QuPath (Fig. 1a). This modular design requires minimal fine-tuning, limited primarily to the YOLO step, and enables robust detection, segmentation, and tracking (i.e., SWIFT core), allowing downstream quantitative analysis. We designed the classification to be optional, modular, and retrained with each dataset as organoid classes vary greatly depending on the applications.

To ensure versatility and model-agnostic performance, we first fine-tuned the YOLOv8s model on a small yet highly diverse dataset of 417 brightfield images of mouse colon organoids, encompassing variations in culture medium, timepoint, resolution, and brightness. Despite the limited training set, the model achieved strong baseline performance (precision = 0.67, recall = 0.83, mAP50 = 0.76). Our fine-tuned model (model A) was then evaluated on an independent dataset, yielding excellent detection metrics (recall = 0.92, F1.50 = 0.79; Fig. 1b—Left panel). To further improve detection fidelity, we implemented an optional quality-control (QC) step to exclude incomplete organoids located at image edges (Fig. 1b—Right panel, Supplementary Video 1). Overall, SWIFT core showed remarkable robustness across users with different levels of expertise (Fig. 1c and Supplementary Fig. 1a).

To further assess robustness and generalizability, we applied SWIFT core to two publicly available organoid datasets acquired in independent laboratories using distinct microscopes. These organoids were derived from four organs (colon, small intestine, lung, and pancreas), and both healthy and tumoral contexts, including cystic fibrosis and pancreatic ductal adenocarcinoma. While these public resources included multiple patients for each organoid type, the number of donors was not consistent. We can confidently state that our workflow was benchmarked on at least 4 donors and 2 species. Without additional fine-tuning, the fine-tuned model A maintained strong performance on human pancreatic ductal adenocarcinoma (PDAC) and lung organoid datasets (Fig. 1d, e, left panels). For datasets with more complex morphologies, such as mouse small intestine and human rectum organoids, the performance declined slightly (Fig. 1d, e, left panels Supplementary Fig. 1b, c) but was rapidly restored by fine-tuning with very few additional annotations (18 organoids for small intestine (model B), 153 for human rectum (model C); (Supplementary Fig. 1b). Only minutes of manual annotation (Supplementary Fig. 1b, c) were sufficient to recover high precision and recall (Fig. 1d, e, right panels, SI and rectum), confirming that organoid detection using SWIFT is both microscope- and organoid-agnostic and can be readily adapted with minimal effort, unlike most existing pipelines (Supplementary Fig. 1d and Supplementary Fig. 2a–d). Following organoid detection, where objects are identified by bounding boxes, SWIFT uses these regions as inputs for SAM-based segmentation. We evaluated segmentation performance by comparing manual annotations (QuPath brush tool), semi-automated segmentation (SAM integrated in QuPath), and the fully automated SWIFT pipeline. This analysis demonstrates that SWIFT achieves high-fidelity segmentation comparable to expert annotations (Supplementary Fig. 1e).

When benchmarked against the original methods associated with each publicly available dataset described above, SWIFT performed similarly or outperformed previously reported approaches (Supplementary Fig. 2a–d), with particularly strong gains in segmentation efficiency (Supplementary Fig. 2d).

### Spatiotemporal resolution of individual objects for quantitative analysis of phenotypic dynamics in organoid cultures

We next focused on the analysis of intestinal organoids, a widely used in vitro model to study epithelial homeostasis, regeneration, and disease. In order to train our application-specific classifier, we used four thoroughly described canonical intestinal morphologies, cystic, columnar, budding, and dying, based on histological (Supplementary Fig. 3a), morphological features (Fig. 1f) and molecular validations^5,13,14^ (Supplementary Fig. 5). Using parameters such as area, circularity, mean intensity, intensity standard deviation (SD), these distinct phenotypes can be reliably distinguished in brightfield microscopy (Fig. 1f–h, Supplementary Fig. 3b, c). Feature extraction and supervised Random Tree object classification in QuPath enabled efficient discrimination of the four morphological phenotypes (Fig. 1g, h). While cropped organoids (Supplementary Fig. 3d) were assigned to a dedicated class (i.e., Ignore) to be excluded from the analysis, our mouse intestinal organoid-specific classifier achieved excellent performances in recall (> 0.94) and F-1 score (> 0.95) (Supplementary Fig. 3e). To capture dynamic transitions, we implemented a centroid-based nearest-neighbor tracking algorithm (Supplementary Fig. 4) that links individual organoids across time-lapse frames within a 150 µm displacement threshold. This threshold was chosen based on quantifying the organoid displacement between consecutive days in the analyzed timelapses (Supplementary Fig. 4c). This approach reconstructs multi-day lineage trajectories that capture both morphological features and transitions (e.g., cystic-to-budding), providing dynamic single-organoid profiles that complement static population-level analyses (Supplementary Video 2).

### Live organoid tracking of Wnt-dependent transitions between regenerative and homeostatic epithelial states

**Fig. 2:**
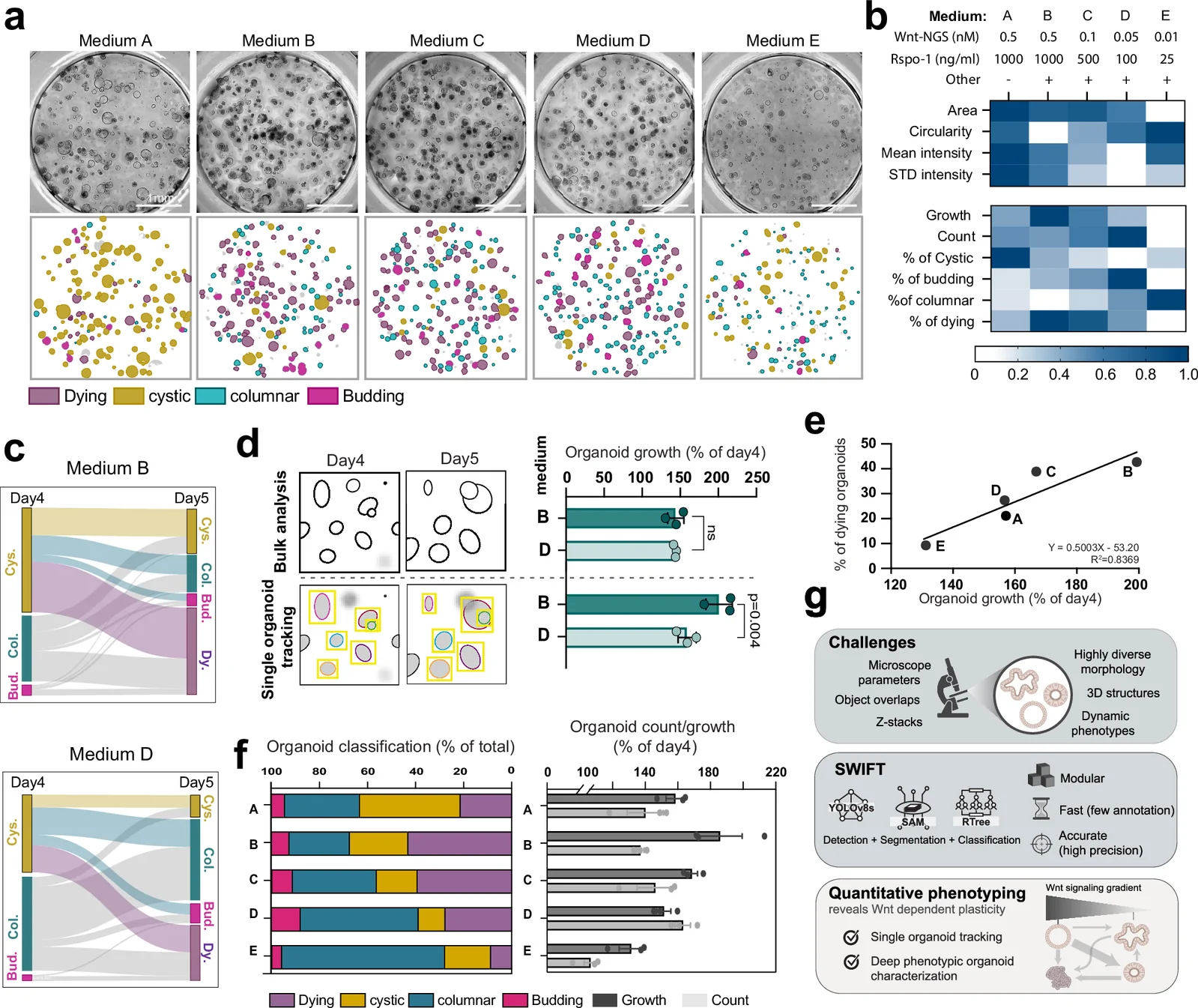
Quantitative phenotyping reveals Wnt-dependent plasticity in mouse colon organoids. **a** Imaging and SWIFT annotation of colon organoids under different culture conditions. Representative images of *n* = 3 replicates per condition. Scale bar = 1 mm. **b** Heatmap of row-normalized features (upper panel), growth and phenotypes (lower panel) of colon organoids cultured in different media compositions for 5 days. *n* = 3 replicates per condition were quantified and averaged in the heatmap. **c** Sankey plot representation of phenotypic transition under representative media (B, D) between day4 and day5. **d** Bulk growth analysis vs single-organoid growth analysis (mean ± SEM) after 5 days of culture in medium B and D; two-way ANOVA with Sidak correction was performed and each dot represents an experimental replicate. *n* = 3 replicates per condition were quantified. **e** Correlation between growth rate and organoid death in colon organoids cultured for 5 days in medium A to E. Each dot corresponds to the mean of *n* = 3 replicates. Linear regression with R². **f** Summary of organoid growth, counts, and phenotype fractions (mean ± SEM) across media A–E. *n* = 3 replicates per condition were quantified. **g** Schematics of the present study: Organoid heterogeneity and temporal dynamics hinder quantitative analysis (upper panel). SWIFT integrates YOLO, SAM, and QuPath into a fast, modular pipeline for robust detection, segmentation, classification, and tracking (middle panel). This approach overcomes model-specific limitations, enabling precise, time-resolved phenotypic analysis of organoid growth and Wnt-dependent morphological transitions (lower panel). Icons were created using BioRender.

Organoid culture conditions must be carefully tuned depending on the biological question. To illustrate the analytical power of SWIFT, we quantitatively assessed how varying the concentrations of Wnt and its co-activator R-spondin1 in the culture medium affect the growth and morphology of mouse colon organoids (Fig. 2a, b). We tested a range of formulations (Media A–E) containing different levels of Next-Generation Surrogate Wnt (Wnt-NGS)^15^ and R-Spondin 1 (Media A to E), starting from high Wnt concentrations similar to the conventional WENR medium^3,16^.

In Medium A (WENR), organoids remained large and cystic representative of a proliferative, unpolarized “regeneration-like” epithelium typically observed after intestinal damage (Fig. 2a, b, Medium A)^5^. While this state may be interesting to understand intestinal lamina repopulation, it fails to model intestinal plasticity in which differentiated cells can de-differentiate to re-populate the stem cell niche. An optimized formulation, supplemented in FGF2, IGF1, and NRG1^17,18^ promoted differentiation and maintained stem cell niche features, but also led to accelerated growth and extensive organoid death after five days in culture (Fig. 2a, b, Medium B). Stepwise reduction of Wnt and R-spondin1 progressively decreased cell death while maintaining growth and enriching for differentiating phenotypes (i.e., columnar/budding morphologies), consistent with a “homeostatic-like” epithelium (Fig. 2a, b, Medium C-E). At subthreshold Wnt levels, both organoid growth and survival were severely impaired (Fig. 2a, b, Medium E).

Single-organoid tracking provided insights beyond bulk measurements. Although population-level analyses revealed no significant difference in mean organoid area between Media B and D (Fig. 2d), individual trajectories showed that growth rates correlated with Wnt concentration (Fig. 2e), and rapidly expanding organoids tended to collapse earlier (Fig. 2f, Supplementary Fig. 5a). Analysis of phenotypic transitions (Fig. 2c) further revealed that, under high Wnt conditions, most organoids transitioned to a dying state by day 5, whereas reducing exogenous Wnt favored cystic-to-columnar or cystic-to-columnar or budding transitions, indicative of a return toward a homeostatic-like state. Altogether, this analysis demonstrates a strong correlation between Wnt dosage and organoid growth, survival, and morphology (Fig. 2g). Importantly, these conclusions, derived from SWIFT-based morphological analysis (Fig. 2f, Supplementary Fig. 5a), are supported by independent molecular validations (Supplementary Fig. 5b, c), including qPCR and immunohistological assessment of established lineage and apoptosis markers, reinforcing the relevance of using morphology as a rapid and informative proxy for underlying biological mechanisms.

In summary, SWIFT provides a fast, robust, accurate, and modular solution for time-resolved organoid phenotypic quantification. By integrating YOLOv8s, SAM, and a downstream classification and tracking module, it overcomes key limitations of existing deep learning approaches that require extensive training and expert intervention. SWIFT enables quantitative, dynamic monitoring of organoid behavior and phenotypic transitions (Fig. 2g). Its adaptability makes it particularly well-suited for medium- to high-throughput applications, including drug screening on disease-modeling organoids such as those for cystic fibrosis.

## Methods

### Ethical compliance

We have complied with all relevant ethical regulations for animal use. All animal procedures were performed according to protocols approved by the Veterinary Authorities of the Canton Vaud and according to the Swiss Law (License VD 3497, EPFL) and were in accordance with the ARRIVE guidelines and 3R principle (Reduction, Replacement, Refinement) for laboratory animals.

### Organoids extraction and culture

Colonic tissues from 8-week-old female wild type C57Bl6/J mice were collected after euthanasia with a lethal dose of sodium pentobarbital (150 mg/kg)^19^. First, underlying fat, muscularis and remaining blood vessels were carefully removed from the obtained colons using forceps. The cleaned mucosal tissues were then thoroughly washed on the luminal and basal side with 1X PBS supplemented with antibiotics; 100 U/mL penicillin/streptomycin (Thermo Fisher) and 50 μg/mL primocin (Invivogen). The colons were then cut longitudinally and opened to access the epithelium. The differentiated epithelium was carefully scraped and flushed away with a lamelle in order to only keep the crypts. The tissues were then cut into 1–2 mm pieces and digested enzymatically^20^. The resulting crypts were then washed thoroughly with cold Base Medium (advanced DMEM/F-12 (Thermo Fisher) containing 1 mM HEPES (Thermo Fisher), 1X Glutamax (Thermo Fisher), 100 U/mL penicillin/streptomycin (Thermo Fisher) and 50 μg/mL primocin (Invivogen)). They were finally embedded in basement membrane extract hereafter named BME (Matrigel, Corning) and cultured in expansion medium (Medium B; Base Medium supplemented with 1X B27 -VitA (Thermo Fisher), 1 mM N-acetylcysteine (Sigma-Aldrich), 0.5 nM NGS-WNT (produced in-house), 50 ng/mL murine EGF (PeproTech), 100 ng/mL Noggin (produced in-house), 1 μg/mL R-Spondin1 (produced in-house), 0.5 μM A83-01 (Tocris), 100 ng/mL human IGF-1 (Biolegend), 50 ng/mL human FGF-2 (PeproTech), 10 nM human Gastrin (Sigma-Aldrich) and 1 ng/mL human NRG1 (PeproTech))^18^.

Organoids were established within 1–2 weeks and were passaged every 5 days in Medium B. Medium A (WENR) consisted of Base Medium supplemented with 1X B27 -VitA (Thermo Fisher), 1 mM N-acetylcysteine (Sigma-Aldrich), 0.5 nM NGS-WNT (produced in-house), 50 ng/mL murine EGF (PeproTech), 100 ng/mL Noggin (produced in-house), 1 μg/mL R-Spondin1 (produced in-house). Media C-E consisted in Medium B with varying concentrations of NGS-WNT and R-Spondin1 as described in Fig. 2b. Organoids between passages 5 and 20 were used for this study and were cultured in various culture wares, namely conventional 24-well plates (Corning) for routine cultures or low-throughput assays and alternatively in organoid-specific 96-well plates (Organoid Culture Plates, Stem Cell Technologies) for screening.

### Histological studies

Organoids were washed with PBS1X and fixed in 4% paraformaldehyde overnight at 4 °C, embedded in paraffin and sectioned at 4 µm thickness. Sections were deparaffinized with xylene and rehydrated with distilled water through a series of graded alcohol. Tissues were stained with hematoxylin and eosin (H&E), Alcian blue using standard protocol, and Cleaved Caspase-3 antibody, Cell Signaling Cat no: #9661, according to manufacturer’s instructions (1:400 dilution). Brightfield images were acquired on an Olympus VS200 whole slide scanner using OlyVIA software and ×40 air objective.

### qPCR

Organoids were washed once with PBS, disrupted manually in cold PBS, followed by RNA extraction using RNA easy mini extraction kit (Qiagen). RNA concentration was measured by spectrometry and total RNA was used for reverse transcription. We then used a 1/10 dilution of the cDNA to perform quantitative real-time PCR using SYBR MasterMix (Life Technology) on a Quantstudio 6 Real-Time PCR System (Applied Biosystems). mRNA level in triplicate was normalized using Ribosomal protein S9 (RPS9), Glyceraldehyde 3-phosphate dehydrogenase (GAPDH), and Eukaryotic Translation Elongation Factor 1 Alpha 1 (EEF1A1), and Results were expressed as 2^(−DDCt)*100%. Primers (5’-3’) used are:

**Table:** 

**Gene**	**Forward**	**Reverse**
*mLgr5*	ATTCGGTGCATTTAGCTTGG	CGAACACCTGCGTGAATATG
*mMuc2*	TCCTGACCAAGAGCGAACAC	ACAGCACGACAGTCTTCAGG
*mAlp1*	GCCTATCTCTGTGGGGTCAA	TTTCTTGGCACGGTACATCA
*mGapdh*	ATCCTGCACCACCAACTGCT	GGGCCATCCACAGTCTTCTG
*mEef1a1*	TCCACTTGGTCGCTTTGCT	CTTCTTGTCCACAGCTTTGATGA
*mRps9*	GACCAGGAGCTAAAGTTGATTGGA	TCTTGGCCAGGGTAAACTTGA

### Organoids brightfield image acquisition

Brightfield imaging was performed on organoid cultures at the indicated time. Organoids were imaged on an inverted microscope (Nikon Ti2) equipped with transmitted light (DIA LED) and a 10× objective (Plan Apo λD 10×/0.45 NA, OFN25, DIC N1; objective code MRD70170). Acquisition parameters were: exposure time 4 ms, binning 1 × 1, and full-frame readout. Pixel size at the specimen plane was calibrated to 0.685978 µm px⁻¹ (zoom 1.00×). For each well, a multi-tile brightfield mosaic (2 × 2 tiles) was acquired with 10% tile overlap and software stitching enabled. Z-stacks were acquired using 50 µm axial steps. All imaging was performed with the same exposure, gain, pixel size, and optical configuration across samples to ensure quantitative comparability.

### External brightfield datasets

Publicly available brightfield organoid images were incorporated from two peer-reviewed sources to broaden morphological diversity as well as organoid types: (i) OrgaSegment^7^ and (ii) OrganoID^21^. These datasets include organoids derived from human colon, human PDAC, human lung, mouse small intestine, and human rectum.

### File conversion, data management, and Z-projection

Raw ND2 files were converted to OME-TIFF using the NGFF Converter and uploaded to the institutional OMERO server (EPFL, omero-server.epfl.ch). Image stacks were processed in Fiji via a custom Groovy script (EPFL BIOP; Guiet & Dornier, BSD-3 license) interfacing with OMERO through *simple-omero-client* (v5.9.2). Extended depth-of-focus (EDF) projections were performed on GPU using CLIJ2 with a variance-based projection method (σ = 50). Resulting images were exported as pyramidal OME-TIFFs (LZW compression, full resolution) via the Kheops exporter and re-imported into OMERO for storage and downstream analysis.

### Yolo fine-tuning for organoid detection

To develop a robust detection model across organoid culture conditions, we fine-tuned a YOLOv8s network (Ultralytics v8.3.156) for brightfield organoid detection. The training dataset consisted of extended-depth-of-focus (EDF) images generated from organoids cultured in diverse media conditions and at multiple days of growth, thereby covering a wide morphological range (≈ 10 µm to several hundred µm). To enhance model generalizability, images used for the training set were acquired with different exposure times and included a variety of native resolutions (ranging from 512 × 512 to 7344 × 7344 pixels – pixel size = 0.69 × 0.69).

Ground-truth annotations were generated in QuPath (v0.5.1) by two independent annotators, who delineated organoids using bounding boxes. To maintain consistency, organoids located at image edges or below a minimal size threshold were not annotated. This yielded 417 annotated organoid instances across 16 images, which were split into a training set (*n* = 9) and a validation set (*n* = 7).

Training was performed in Python 3.10.18 and PyTorch 2.7.1 on an Apple M1 Pro CPU. The YOLOv8s model comprised 129 layers with 11,135,987 trainable parameters (28.6 GFLOPs). Training was conducted for 70 epochs with a single-class configuration and a fixed input size of 1024 × 1024 pixels. Data augmentation was enabled with RandAugment, providing stochastic transformations during training. Optimization was carried out with AdamW (initial learning rate = 0.002, momentum = 0.9).

After 70 epochs, the best-performing weights (modelA.pt) achieved the following on the validation set: precision = 0.674, recall = 0.832, mAP50 = 0.762, and mAP50–95 ≈ 0.537. Inference speed was ~558.5 ms per image (CPU).

### Model performance evaluation

The detection performance of the fine-tuned YOLOv8s model (modelA) was evaluated in QuPath (v0.5.1) using a custom Groovy script. Manual ground-truth annotations and YOLO detections were compared on a per-object basis. For each predicted bounding box, the Intersection-over-Union (IoU) was computed with all ground-truth annotations.

Predictions were classified as true positives (TP) if the IoU exceeded a predefined threshold of 0.5. Predictions without a match were counted as false positives (FP), while ground-truth objects not detected by YOLO were counted as false negatives (FN).

From these counts, standard object detection metrics were derived:

Precision: proportion of predicted organoids that were correct: Precision=TP/(TP + FP).

Recall: proportion of annotated organoids that were detected: Recall=TP/(TP + FN).

F1-score: harmonic mean of precision and recall: F1 = 2 × (Precision × Recall)/(Precision + Recall)

mAP (mean Average Precision): approximated from the precision at IoU = 0.5 (mAP@0.5).

To assess robustness across overlap criteria, performance was further evaluated over a range of IoU thresholds (0.30–0.95, step 0.05). At each threshold, TP, FP, FN, precision, and recall were recorded, and the mAP@[0.30:0.95] was approximated as the mean precision across thresholds.

### Automated organoid detection and segmentation

Automated detection and segmentation were carried out on the institutional workstation BIOP Desktop 21 (EPFL BioImaging & Optics Platform). The workstation was equipped with an AMD Ryzen Threadripper PRO 3975WX CPU (32 cores, 64 threads), 256 GB RAM, and an NVIDIA RTX A6000 GPU (48 GB VRAM), providing sufficient computational resources for deep learning inference and interactive image analysis.

Organoid detection was first performed using a fine-tuned YOLOv8 model (modelA.pt) integrated into QuPath v0.5.1. For each field of view, the rendered brightfield image was exported and processed with YOLO to generate bounding-box detections, which were re-imported into QuPath as annotation objects. Each bounding box was subsequently refined using the Segment Anything Model (SAM) via the Elephant-SAM QuPath client connected to a local inference server running on the same workstation. The ViT-L backbone was used, and when multiple segmentation proposals were returned, the best-quality mask was systematically selected to ensure accurate delineation of organoid contours. To guarantee consistent scaling across all images, the viewer magnification was explicitly set to a downsample factor of 1 prior to each SAM prediction, ensuring segmentation at native magnification. YOLO generates overlapping bounding boxes when detecting overlapping organoids. SAM generates masks based on the object contours. If an organoid overlaps above another organoid the front organoid will be segmented entirely and the bottom organoid will be cropped. These objects can be excluded or included, depending on the type of analysis, using the classifier (see below).

Resulting masks were stored as polygonal annotations and all annotations were synchronized with the institutional OMERO server for centralized storage and downstream quantitative analysis.

### Organoid classifications

For each segmented organoid, both morphometric measurements (area, perimeter, circularity, solidity, maximum and minimum diameter) and intensity-based features (mean, standard deviation, minimum, maximum, and median pixel intensities) were extracted from the brightfield channel at 2.00 µm/pixel resolution. These features were used to train a supervised object classifier in QuPath v0.5.1. The classifier was implemented as a Random Trees (RTrees) model through the OpenCV ML framework, configured as a multi-class classifier with 50 decision trees, a maximum depth of 25, and a minimum sample size of 10 objects per split. Surrogate splits and cross-validation were disabled, and categorical and ordinal variables were internally encoded by QuPath.

Between 100 and 200 manually annotated organoids were used as training examples, for each of the five morphological categories (cystic, columnar, budding, dying, or ignore) defined during manual annotation. The resulting model showed negligible out-of-bag error within the training dataset. Our classifier assigns organoids with cropped masks (see section “Automated organoid detection and segmentation”) to the Ignore class. This class was excluded from the phenotypic analysis.

### Single-organoid tracking across consecutive days

Tracking of individual organoids across consecutive imaging days was performed in R using a centroid-proximity, one-to-one linking strategy. Measurements and metadata were read from QuPath exports (.csv), with processing carried out using the packages readr, dplyr, stringr, tidyr, purrr, and openxlsx.

### Link hypothesis generation and gating

For each segmented object, image metadata were used to extract the acquisition timepoint, experimental condition (up to five tokens), a unique object identifier, the classifier-assigned label, centroid coordinates, and associated morphometric and intensity features. Within each condition, all possible object pairs between consecutive acquisitions were generated; for longer-term tracking, this procedure was repeated independently for each interval. For each candidate pair, the Euclidean distance between centroids was calculated as follows: d = √((xj−xi)2 + (yj−yi)2).

Pairs with a centroid distance ≤150 µm were retained as candidate links. Because organoid plates were not continuously imaged but repositioned on the microscope stage each day, the threshold was intentionally set to accommodate modest translational offsets introduced by plate repositioning in addition to true biological movement while minimizing spurious associations.

### Greedy one-to-one assignment

Candidate links were ranked by increasing distance within each field. A greedy nearest-neighbor assignment was then applied, enforcing uniqueness such that no object could be linked more than once. This produced the best links across each day-to-day transition. Tracks were then chained across multiple consecutive days by merging through shared intermediate identifiers, allowing trajectories to span the full experimental timeline. Tracks could terminate early if no valid continuation was available (e.g., object disappearance or failure to meet distance criteria).

For each linked object, all per-day attributes were merged back into the trajectory, including morphometric and intensity measurements (area, length, circularity, solidity, maximum and minimum diameter; and mean, SD, min, max, median intensity), centroid coordinates, and classifier-assigned classes.

### Sankey plot visualization

Sankey diagrams were generated in R (packages: dplyr, tidyr, networkD3, htmlwidgets, webshot) to visualize organoid class transitions across consecutive timepoints. Each tracked organoid was represented by its assigned class at successive acquisitions, and flows were computed as counts of objects moving from one class to another. Nodes corresponded to all observed classes at each timepoint, while link widths reflected the frequency of transitions.

### Statistics and reproducibility

All statistical analyses were conducted using GraphPad Prism v10.2.2 (GraphPad Software). Details regarding data visualization, statistical tests, and sample sizes are provided in the corresponding figure legends. Data distributions were assessed for normality using the Shapiro–Wilk test, and either parametric or non-parametric statistical tests were applied as appropriate. For ANOVA analyses, multiple-comparison corrections were performed using Tukey’s or Sidak’s post hoc tests, as indicated. Sample sizes were established based on previous experimental observations and power calculations to provide sufficient statistical power. Data variability is presented as standard error of the mean, and group variances were comparable for all statistical comparisons performed. Experimental groups were randomized, and investigators remained blinded to group assignment throughout data acquisition and analysis. All samples meeting predefined quality-control criteria were included in the analyses, with exclusions restricted to imaging or segmentation artifacts. Biological replicates, technical replicates, and sample sizes are defined in the corresponding figure legends. Statistical significance was defined as *P* < 0.05.

### Use of artificial intelligence tools

ChatGPT (OpenAI, GPT-5) was used under author supervision to assist in generating and debugging R scripts for data processing and visualization, as well as for improving the clarity and conciseness of the text. All code and written content were reviewed, verified, and approved by the authors.

### Reporting summary

Further information on research design is available in the Nature Portfolio Reporting Summary linked to this article.

## Supplementary information

Transparent Peer Review file

Supplementary Information

Description of Additional Supplementary Files

Supplementary Data 1

Supplementary Video 1

Supplementary Video 2

Reporting Summary

## Data Availability

All source data is available in the online version of this article. Source data can be obtained from Supplementary Data 1.

## Code availability

All imaging data are hosted on the EPFL institutional OMERO server (https://omero.epfl.ch/). External datasets were retrieved from OrgaSegment^7^ and OrganoID^21^. Custom scripts for Fiji (Groovy) and QuPath (Groovy) are available on GitHub (https://github.com/upvdg/SWIFT).
