# Supplementary material for: A Single-organoid Workflow for quantitative Imaging classiFication and Tracking (SWIFT)
Source: Commun Biol. 2026 Sep 1;9:1149. doi: 10.1038/s42003-026-10768-x (PMC13534601; doi:10.1038/s42003-026-10768-x)
Supplement: Supplementary file 1 [file 42003_2026_10768_MOESM1_ESM.pdf]

nature portfolio

Peer Review File

A Single-organoid Workflow for quantitative Imaging classiFication and Tracking (SWIFT)

Corresponding Author: Dr Nathalie Brandenberg

This file contains all reviewer reports in order by version, followed by all author rebuttals in order by version.

Version 0:

Reviewer comments:

Reviewer #1

(Remarks to the Author)
In this manuscript, Bracq et al developed a method of 'SWIFT' referred as single-organoid workflow for quantitative imaging classiFication and Tracking. They declare it as a fast and modular pipeline for quantitative, time-resolved organoid analysis from brightfield images across organoid types and microscope systems. They also applied it to intestinal organoids and uncovers dynamic Wnt-dependent morphological transitions, enabling scalable, high-throughput studies of epithelial plasticity. Overall, they provide possible strategies for quantitatively analyzing of in vitro organoid in a fast versatile and scalable level. However, the study lack the basic information of numbers of image and donors in clinic.
Major concern
1.Although four types of morphology as cystic, budding, columnar and dying was defined, we usually get more shapes in clinic from different donors. Is this classification cover all types of organoids? Also, how many donors are those organoids derived from for different tissues?
2.How this 'SWIFT' method make sure the accuracy in analyzing patterning overlapping of organoids. Single organoids from different layers?
3.Has pathological condition been included and analyzed ?

Reviewer #2

(Remarks to the Author)
This manuscript by Lucie et al. introduces SWIFT, a modular workflow integrating YOLOv8 detection, SAM segmentation, and QuPath-based classification and tracking for brightfield organoid analysis. The pipeline performs across multiple organoid systems and requires minimal annotation, and the authors illustrate its utility through a case study on Wnt-dependent morphological transitions. The work is timely, well-written, and addresses a genuine need for scalable organoid imaging analysis, but several methodological gaps limit the strength of the conclusions. Key benchmarking against existing tools is lacking. Segmentation, classifier, and tracking accuracy should be further validated. Finally, the biological conclusions rely exclusively on morphology and require molecular validation of apoptosis, proliferation, and differentiation states. These issues do not undermine the concept but indicate the need for more rigorous evidence. Followings are the reviewer's recommendation to be revised.

Major Points
1. Quantitative benchmarking (Pages 2&8): Although YOLO detection is validated, SAM-based segmentation and the tracking module lack quantitative evaluation, which is essential for confidence in downstream phenotype identification. It is necessary to evaluate segmentation accuracy using manually segmented ground truth (e.g. F1, Dice score, IoU).

2. Classifier robustness and generalizability (Pages 2&9): The Random Trees classifier is trained on 100–200 organoids per class, yet the manuscript presents only internal validation, leaving the robustness and generalizability of the model insufficiently demonstrated. A more rigorous validation strategy should be implemented, such as using a synthetic/publicly available dataset, which would provide a clearer assessment of the classifier's ability to generalise beyond the training data. In addition to improved validation design, the manuscript would benefit from reporting class-specific performance metrics, including precision, recall, and F1-scores, to allow readers to evaluate how well the classifier distinguishes among the cystic, round, budding, dying, and "ignore" phenotypes.

3. Comparison to existing pipelines under identical conditions (Page 2): The manuscript currently provides only a qualitative comparison of existing organoid analysis pipelines in Supplementary Figure 1f (page 14), which does not allow readers to evaluate SWIFT's performance relative to established methods. A direct, quantitative benchmark is essential. The authors should therefore compare SWIFT against all or some of the latest reported pipelines using the same dataset under identical imaging and preprocessing conditions. For each tool, key performance indicators should be reported, including segmentation accuracy measured by Intersection-over-Union (IoU), detection performance quantified through precision, recall, and mean Average Precision (mAP), and computational efficiency represented by runtime per image or per dataset. In addition, the comparison should document the annotation burden required for each method, since the claim that SWIFT minimises manual annotation is central to its proposed contribution.

4. Biological validation (Page 3): The current analysis shows that SWIFT detects an increased fraction of dying organoids under high-Wnt conditions (medium B, Fig. 2) and a shift toward columnar and budding morphologies upon Wnt reduction (medium D, Fig. 2), but these conclusions remain purely morphological (in Supplementary Figure 2a) and are not supported by independent biological readouts. To substantiate the claim that these phenotypic classes reflect bona fide cell states, molecular validations using established protein markers should be examined. In high-Wnt media, staining for apoptosis markers such as cleaved caspase-3 would confirm that organoids classified as "dying" indeed undergo cell death. Conversely, in medium D for example, immunostaining or in situ hybridisation for stemness and differentiation markers (e.g. Lgr5 for stem cells, Muc2 for goblet cells, and Alpi for intestinal epithelium) would demonstrate that "columnar" and "budding" morphologies correspond to more differentiated or niche-like epithelial states.

5. Tracking assumptions (Pages 4&9-10): The current tracking strategy relies on a fixed centroid distance threshold of 150–200 µm to link organoids across timepoints, but this choice is neither biologically nor technically justified in the manuscript, leaving uncertainty about the robustness of the trajectories. To justify this, the workflow should first provide a distribution of organoids across all datasets, illustrating how far organoids typically move between acquisitions and thereby motivating the selected threshold.
In addition, they should demonstrate that tracking performance is robust to reasonable variation in this parameter by comparing key metrics such as tracking, detection and lineage accuracy across multiple threshold values.
Finally, although fusion or splitting events may be rare, the pipeline's behaviour in such scenarios should be explicitly illustrated, for example, by showing representative cases where organoids merge or divide and demonstrating that the linking logic assigns identities in a biologically plausible manner.

Minor Points
1. Figure 1c shows multiple types of organoids processed with multiple SWIFT models. For better comparison, include panels of every organoid type processed with every model.
2. Figure 2g is mislabelled in the legend.
3. Figure 2b, the colour palette of the heatmap could be visually improved by avoiding a hue gradient.
4. Supplementary Figure 1g-h needs clearer statistical annotations; error bars are hard to see.
5. Supplementary Figure 1g-h is mislabelled in the legend.
6. On page 2, in the third paragraph, "For datasets with more complex morphologies, such as mouse small intestine and human rectum organoids, the performance declined slightly (Supplementary Fig. 1b–c)" should change to "(Supplementary Fig. 1c–d)"
7. On page 3, in the second paragraph, "Respond-1" is possibly a typographic mistake
8. On page 4, in the second paragraph, "Fig. 2h" should change to "Fig. 2g"
9. On page 9, the phenotype "Round" appears only in the classification model, not in the main text. This inconsistency should be addressed.

Reviewer #4

(Remarks to the Author)
I co-reviewed this manuscript with one of the reviewers who provided the listed reports. This is part of the Communications Biology initiative to facilitate training in peer review and to provide appropriate recognition for Early Career Researchers who co-review manuscripts.

Version 1:

Reviewer comments:

Reviewer #2

(Remarks to the Author)
All the concerns raised by this reviewer have been addressed appropriately.

Reviewer #4

(Remarks to the Author)
I co-reviewed this manuscript with one of the reviewers who provided the listed reports. This is part of the Communications Biology initiative to facilitate training in peer review and to provide appropriate recognition for Early Career Researchers who

co-review manuscripts.

Open Access This Peer Review File is licensed under a Creative Commons Attribution 4.0 International License, which permits use, sharing, adaptation, distribution and reproduction in any medium or format, as long as you give appropriate credit to the original author(s) and the source, provide a link to the Creative Commons license, and indicate if changes were made.
In cases where reviewers are anonymous, credit should be given to 'Anonymous Referee' and the source.
The images or other third party material in this Peer Review File are included in the article’s Creative Commons license, unless indicated otherwise in a credit line to the material. If material is not included in the article’s Creative Commons license and your intended use is not permitted by statutory regulation or exceeds the permitted use, you will need to obtain permission directly from the copyright holder.
To view a copy of this license, visit https://creativecommons.org/licenses/by/4.0/

Manuscript number: COMMSBIO-25-11082-T

Corresponding author(s): Nathalie Brandenberg

All revised text in the revised manuscript is written in blue, highlighted in gray.

Reviewers' comments:

Reviewer #1 (Remarks to the Author):

In this manuscript, Bracq et al developed a method of 'SWIFT' referred as single-organoid workflow for quantitative imaging classiFication and Tracking. They declare it as a fast and modular pipeline for quantitative, time-resolved organoid analysis from brightfield images across organoid types and microscope systems. They also applied it to intestinal organoids and uncovers dynamic Wnt-dependent morphological transitions, enabling scalable, high-throughput studies of epithelial plasticity. Overall, they provide possible strategies for quantitatively analyzing of in vitro organoid in a fast versatile and scalable level. However, the study lack the basic information of numbers of image and donors in clinic.

Major concern

1. Although four types of morphology as cystic, budding, columnar and dying was defined, we usually get more shapes in clinic from different donors. Is this classification cover all types of organoids? Also, how many donors are those organoids derived from for different tissues?

COMMON REPLY TO Reviewer #1, Major concern 1. and Reviewer #2, Major Point 2.

We thank both reviewers for their essential comments. While the core of SWIFT uses YOLO-based detection and SAM-based segmentation that are microscope- and organoid-agnostic, the classification layer is application-specific. Therefore, the phenotypic classification is an optional downstream module implemented in QuPath and is intentionally not designed to be universal for organoid types and microscopes. Instead, the classifier is meant to be retrained for each biological context and imaging configuration, as conventionally done in supervised morphological annotation. In order to clarify this point, we revised the Abstract and the Main text (page1, line 12 and page 2, lines 49-52) to reflect this particularity of our workflow.

In the present study, we focused on four canonical intestinal morphologies, cystic, columnar, budding and dying, that are widely reported in the literature and appropriate for interrogating Wnt-dependent epithelial plasticity. These classes were not intended to represent an exhaustive catalogue of clinical organoid morphologies.

Regarding robustness, we agree that the manuscript did not sufficiently distinguish between generalizability of the detection-segmentation core of the workflow and transferability of the classifier. We revised the Main text (page 3, lines 93-94) to explicitly state that the Random Trees classifier was trained and validated within each dataset and that its performance is not assumed to generalize across organoid types without retraining. We also expanded the Main text (page 3, lines 100-103, and Supplementary Figure 3e) to report class-specific precision, recall and F1-scores for the intestinal classifier used in this study, providing a clearer quantitative assessment of classification accuracy.

Concerning donor diversity, SWIFT was benchmarked on in-house mouse colon organoids derived from three independent animals (one representative donor shown in figures) and on publicly available datasets (OrgaSegment and OrganoID). Although these public resources include multiple patients for each organoid type, numbers of donor are not consistently provided, preventing us from reporting exact numbers for all analyses. We revised the Main text detailing tissue origin, species, disease status, microscope type and dataset source for all images analyzed (page 2, lines 67-71). Across all experiments, SWIFT was nevertheless tested on organoids derived from at least four independent donors spanning two species (mouse and human), four organs (colon, small intestine, lung and pancreas), and both healthy and pathological contexts, including cystic fibrosis and pancreatic ductal adenocarcinoma. Details about number of images/organoids for each quantification shown are provided in the corresponding figure legends.

2. How this 'SWIFT' method make sure the accuracy in analyzing patterning overlapping of organoids. Single organoids from different layers?

We thank the reviewer for raising this important aspect. YOLO detects individual organoids even when they partially overlap in projection. In the revised manuscript, we clarified the Main text (page 3, line 101) and included representative examples illustrating typical overlapping cases and corresponding SAM segmentations in the Supplementary Figures (Supplementary Figure 3d). SAM-based segmentation can yield to cropped masks in strongly overlapping cases; this should therefore be handled explicitly at the analysis stage.

Because SWIFT is modular, overlapping or partially segmented objects can be treated in different ways depending on the biological question. For instance, when quantifying organoid numbers, YOLO detections alone are sufficient and overlapping objects are retained. For analyses relying primarily on intensity features (for example, classification of living versus dying organoids), partially segmented objects can still be included. In contrast, for morphology-dependent metrics such as area, circularity or solidity, ambiguous objects can be excluded from the analysis manually or using a Random tree classifier made for this purpose.

We clarified these design choices in the Methods, in section "Automated organoid detection and segmentation" (page 10, lines 348-352) and in section "Organoid classifications" (page 11, lines 370-372).

3. Has pathological condition been included and analyzed ?

Pathological organoids were already included through the publicly available datasets used in this study, notably pancreatic ductal adenocarcinoma and lung cancer organoids, as well as human rectal organoids derived from cystic fibrosis patients. We clarified this more explicitly in the Main text of the revised manuscript (page 2, lines 67-71) describing the datasets analyzed, specifying tissue origin, disease status, donor source when available.

Reviewer #2 (Remarks to the Author):

This manuscript by Lucie et al. introduces SWIFT, a modular workflow integrating YOLOv8 detection, SAM segmentation, and QuPath-based classification and tracking for brightfield organoid analysis. The pipeline performs across multiple organoid systems and requires minimal annotation, and the authors illustrate its utility through a case study on Wnt-dependent morphological transitions. The work is timely, well-written, and addresses a genuine need for scalable organoid imaging analysis, but several methodological gaps limit the strength of the conclusions. **Key benchmarking against existing tools is lacking. Segmentation, classifier, and tracking accuracy should be further validated**. Finally, the biological conclusions rely exclusively on morphology and require **molecular validation of apoptosis, proliferation, and differentiation states.** These issues do not undermine the concept but indicate the need for more rigorous evidence. Followings are the reviewer's recommendation to be revised.

Major Points

1. Quantitative benchmarking (Pages 2&8): Although YOLO detection is validated, SAM-based segmentation and the tracking module lack quantitative evaluation, which is essential for confidence in downstream phenotype identification. It is necessary to **evaluate segmentation accuracy using manually segmented ground truth** (e.g. F1, Dice score, IoU).

We thank the reviewer for this relevant remark. We performed manual segmentations (manual) as well as segmentations performed by SAM from manual bounding boxes (semi-automatic) and compared their IoU performances to the ones of our fully automated detection-segmentation, i.e. Yolo-SAM (automatic) workflow. We added in the supplementary data (see Supplementary Figure 1d) the IoU quantifications to determine whether bounding-box initialization influences final contour accuracy. We could demonstrate that except from a few outliers, the IoU is very high >90% between fully manual ground truths, semi-automatic and fully automatic segmentations. To highlight these findings, we modified the Main text on page 3, lines 81-90.

2. Classifier robustness and generalizability (Pages 2&9): The Random Trees classifier is trained on 100–200 organoids per class, yet the manuscript presents only internal validation, leaving the robustness and generalizability of the model insufficiently demonstrated. A more rigorous validation strategy should be implemented, such as **using a synthetic/publicly available dataset**, which would provide a clearer assessment of the **classifier's ability to generalise beyond the training data**.

COMMON REPLY TO Reviewer #1, Major concern 1. and Reviewer #2, Major Point 2.

We thank both reviewers for their essential comments. While the core of SWIFT uses YOLO-based detection and SAM-based segmentation that are microscope- and organoid-agnostic, the classification layer is application-specific. Therefore, the phenotypic classification is an optional downstream module implemented in QuPath and is intentionally not designed to be universal for organoid types and microscopes. Instead, the classifier is meant to be retrained for each biological context and imaging configuration, as conventionally done in supervised morphological annotation. In order to clarify this point, we revised the Abstract and the Main text (page1, line 12 and page 2, lines 49-52) to reflect this particularity of our workflow.

In the present study, we focused on four canonical intestinal morphologies, cystic, columnar, budding and dying, that are widely reported in the literature and appropriate for interrogating Wnt-dependent

epithelial plasticity. These classes were not intended to represent an exhaustive catalogue of clinical organoid morphologies.

Regarding robustness, we agree that the manuscript did not sufficiently distinguish between generalizability of the detection-segmentation core of the workflow and transferability of the classifier. We revised the Main text (page 3, lines 93-94) to explicitly state that the Random Trees classifier was trained and validated within each dataset and that its performance is not assumed to generalize across organoid types without retraining. We also expanded the Main text (page 3, lines 100-103, and Supplementary Figure 3e) to report class-specific precision, recall and F1-scores for the intestinal classifier used in this study, providing a clearer quantitative assessment of classification accuracy.

Concerning donor diversity, SWIFT was benchmarked on in-house mouse colon organoids derived from three independent animals (one representative donor shown in figures) and on publicly available datasets (OrgaSegment and OrganoID). Although these public resources include multiple patients for each organoid type, numbers of donor are not consistently provided, preventing us from reporting exact numbers for all analyses. We revised the Main text detailing tissue origin, species, disease status, microscope type and dataset source for all images analyzed (page 2, lines 67-71). Across all experiments, SWIFT was nevertheless tested on organoids derived from at least four independent donors spanning two species (mouse and human), four organs (colon, small intestine, lung and pancreas), and both healthy and pathological contexts, including cystic fibrosis and pancreatic ductal adenocarcinoma. Details about number of images/organoids for each quantification shown are provided in the corresponding figure legends.

In addition to improved validation design, the manuscript would benefit from reporting class-specific performance metrics, including **precision, recall, and F1-scores**, to allow readers to evaluate how well the classifier distinguishes among the cystic, columnar, budding, dying, and “ignore” phenotypes.

Class-specific performance metrics were calculated according to the definitions provided in the Methods for each classifier category and are now included in the supplementary data (Supplementary Figure 3e).

3. Comparison to existing pipelines under identical conditions (Page 2): The manuscript currently provides only a qualitative comparison of existing organoid analysis pipelines in Supplementary Figure 1f (page 14), which does not allow readers to evaluate SWIFT’s performance relative to established methods. A direct, quantitative benchmark is essential. The authors should therefore compare SWIFT against all or some of the latest reported pipelines using the same dataset under identical imaging and preprocessing conditions. For each tool, key performance indicators should be reported, including segmentation accuracy measured by Intersection-over-Union (IoU), detection performance quantified through precision, recall, and mean Average Precision (mAP), and computational efficiency represented by runtime per image or per dataset.

In addition, the comparison should document the annotation burden required for each method, since the claim that SWIFT minimises manual annotation is central to its proposed contribution.

We thank the review for raising this benchmarking aspect. We thus performed quantitative benchmarking against two published pipelines that provide publicly available image datasets: OrganoID and OrgaSegment and added an entirely new supplementary figure (see Supplementary Figure 2) as

well as amended the Main text highlighting this analysis (page 3, lines 87-90). Because these tools are distributed as pre-trained models optimized for specific imaging conditions and organoid types, applying them directly to our in-house mouse colon brightfield mosaics and EDF projections would not constitute a fair “identical conditions” comparison and could be misleading. Accordingly, in the revised manuscript we conducted head-to-head evaluations on both our internal datasets and the shared public datasets (OrganoID and OrgaSegment images) using identical preprocessing and analysis protocols, and report the requested performance indicators, including precision and recall as well as IoU for segmentation.

In addition, we agree that annotation burden is a central consideration and appreciate the reviewer’s emphasis on this point. Rather than fully retraining each external pipeline from scratch, which would require generating large numbers of pixel-accurate segmentation masks and extensive engineering effort specific to those architectures, we reported and compared annotation requirements as documented in the original publications for each method. For example, OrgaSegment was trained using >15,000 manually segmented organoids across hundreds of images, while OrganoID relied on pixel-level annotations for dozens of training images, in contrast to SWIFT, which required only 417 bounding-box annotations for YOLO fine-tuning (as shown in Supplementary Figure 1e). An important practical distinction is that YOLO fine-tuning relies on bounding-box labels, which are substantially faster to generate and iterate than pixel-level masks, whereas OrgaSegment and OrganoID typically require dense manual segmentations and heavier training setups. We believe that citing these published training requirements provides a fair and reproducible estimate of annotation burden.

4. Biological validation (Page 3): The current analysis shows that SWIFT detects an increased fraction of dying organoids under high-Wnt conditions (medium B, Fig. 2) and a shift toward columnar and budding morphologies upon Wnt reduction (medium D, Fig. 2), but these conclusions remain purely morphological (in Supplementary Figure 2a) and are not supported by independent biological readouts. To substantiate the claim that these phenotypic classes reflect bona fide cell states, molecular validations using established protein markers should be examined. In high-Wnt media, staining for apoptosis markers such as cleaved caspase-3 would confirm that organoids classified as “dying” indeed undergo cell death. Conversely, in medium D for example, immunostaining or in situ hybridisation for stemness and differentiation markers (e.g. Lgr5 for stem cells, Muc2 for goblet cells, and Alpi for intestinal epithelium) would demonstrate that “columnar” and “budding” morphologies correspond to more differentiated or niche-like epithelial states.

In the revised manuscript, we added molecular validations in the supplementary data using both quantitative PCR and immunohistochemical analyses on matched cultures (see Supplementary Figure 5) as well as modified the Main text (page 4, lines 141-146) to provide independent biological evidence that the “cystic”, “dying,” “columnar,” and “budding” phenotypes observed in brightfield correspond to bona fide cellular states. Specifically, we assessed apoptosis in high-Wnt conditions by staining for cleaved caspase-3, and examined stemness and differentiation states under reduced-Wnt conditions using established markers, including *Lgr5* for stem cells, *Muc2* for goblet cell differentiation, and *Alpi* for enterocyte maturation. We confirm that high Wnt media gives rise to more organoids undergoing cell death whereas low organoids grow in low Wnt media express more differentiation or niche-like epithelial markers corresponding to more columnar and budding morphologies.

5. Tracking assumptions (Pages 4&9-10): The current tracking strategy relies on a fixed centroid distance threshold of 150–200 µm to link organoids across timepoints, but this choice is neither biologically nor technically justified in the manuscript, leaving uncertainty about the robustness of the trajectories. To justify this, the workflow should first provide a distribution of organoids across all datasets, illustrating how far organoids typically move between acquisitions and thereby motivating the selected threshold. In addition, they should demonstrate that tracking performance is robust to reasonable variation in this parameter by comparing key metrics such as tracking, detection and lineage accuracy across multiple threshold values.

Finally, although fusion or splitting events may be rare, the pipeline's behaviour in such scenarios should be explicitly illustrated, for example, by showing representative cases where organoids merge or divide and demonstrating that the linking logic assigns identities in a biologically plausible manner.

In the revised manuscript, we performed manual curation of representative time-lapse sequences and quantify the distribution of organoid displacements between consecutive acquisitions across all datasets and added an entirely new supplementary figure (see Supplementary Figure 4). These measurements were used to motivate the centroid-distance threshold applied in this study. The Main text was modified to reflect this motivation (page 3, lines 106-108). Because organoid plates were not continuously imaged but repositioned on the microscope stage each day, the threshold was intentionally set to accommodate modest translational offsets introduced by plate repositioning in addition to true biological movement. We made this experimental constraint explicit in the Methods, section "Link hypothesis generation and gating" (page 11, lines 386-390).

Minor Points

1. Figure 1c shows multiple types of organoids processed with multiple SWIFT models. For better comparison, include panels of every organoid type processed with every model.

2. Figure 2g is mislabelled in the legend.

3. Figure 2b, the colour palette of the heatmap could be visually improved by avoiding a hue gradient.

4. Supplementary Figure 1g-h needs clearer statistical annotations; error bars are hard to see.

5. Supplementary Figure 1g-h is mislabelled in the legend.

6. On page 2, in the third paragraph, "For datasets with more complex morphologies, such as mouse small intestine and human rectum organoids, the performance declined slightly (Supplementary Fig. 1b–c)" should change to "(Supplementary Fig. 1c–d)"

7. On page 3, in the second paragraph, "Respond-1" is possibly a typographic mistake

8. On page 4, in the second paragraph, "Fig. 2h" should change to "Fig. 2g"

9. On page 9, the phenotype "Round" appears only in the classification model, not in the main text. This inconsistency should be addressed.

We thank the reviewer for pointing out these imprecisions. We addressed them all (except from point 3) on the relevant pages.

Reviewer #3 (Remarks to the Author):

I co-reviewed this manuscript with one of the reviewers who provided the listed reports. This is part of the Communications Biology initiative to facilitate training in peer review and to provide appropriate recognition for Early Career Researchers who co-review manuscripts.
