# Supplementary material for: A Single-organoid Workflow for quantitative Imaging classiFication and Tracking (SWIFT)
Source: Commun Biol. 2026 Sep 1;9:1149. doi: 10.1038/s42003-026-10768-x (PMC13534601; doi:10.1038/s42003-026-10768-x)

# SUPPLEMENTARY FIGURE 1

**a**

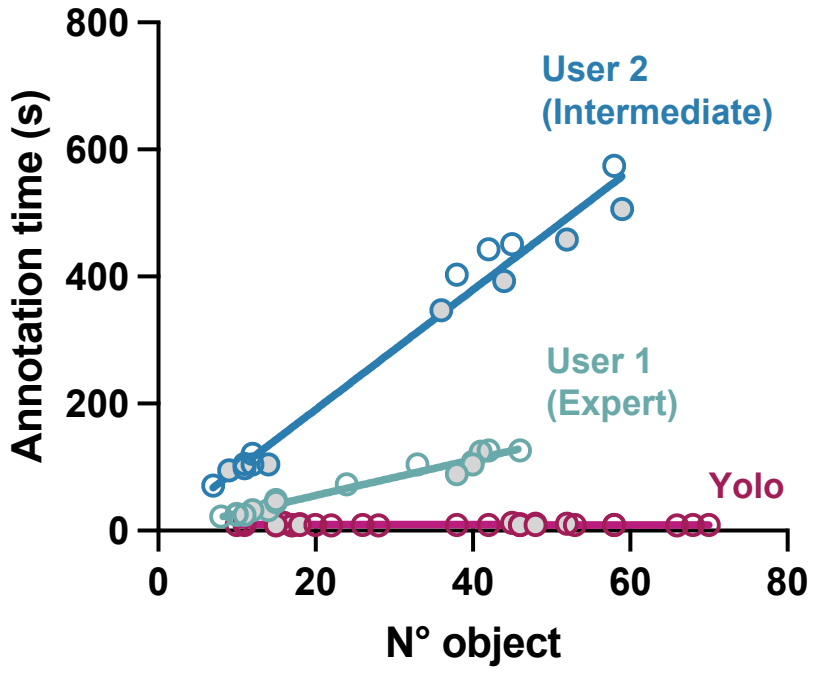


**b**

| | Number of images | | Total number of annotations | Estimated time to annotate (min) | Additional annotations to adjust model | Estimated time to adjust model (min) |
|---|---|---|---|---|---|---|
| | Training | Validation | | | | |
| Model A | 7 | 9 | 417 | 42 | - | - |
| Model B | 9 | 12 | 435 | - | 18 | 2 |
| Model C | 9 | 11 | 573 | - | 156 | 15 |

**c**

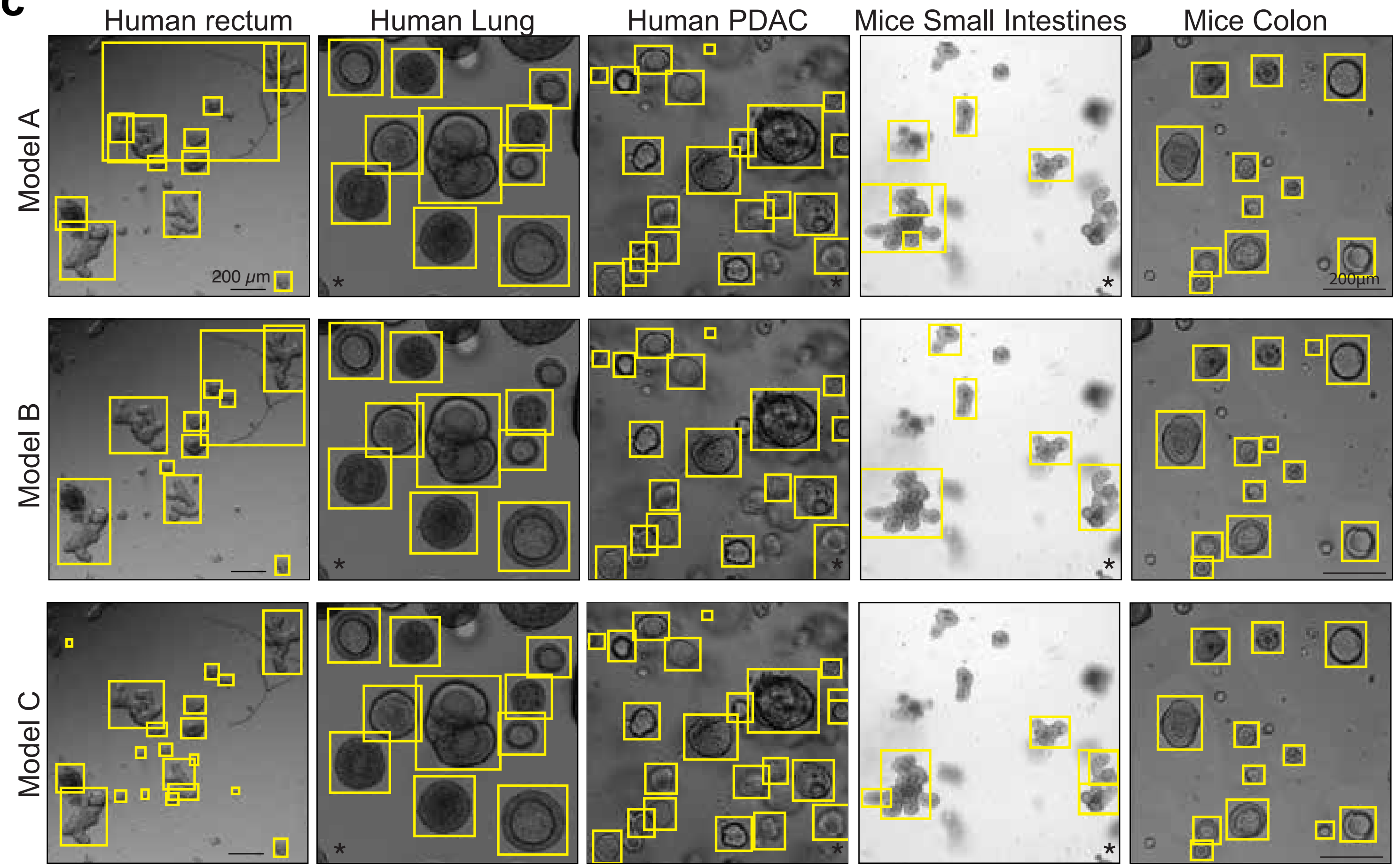


**d**

| Publication | Year | Method | Det. | Seg. | Class. | Nbr Annotations | Organoid specificity | Microscope specificity |
|---|---|---|---|---|---|---|---|---|
| **OrganoSeg** (Borten 2018) | 2018 | Binary | Yes | Yes | No | N/A | Broad | Broad |
| **OrgaQuant** (Kassis 2019) | 2019 | CNN | Yes | No | No | 14,242 (bb[1]) | Specific | Specific |
| **GOAT** (Bremer 2022) | 2022 | CNN | Yes | Yes | No | 90,210 (m[2]) | Broad | Broad |
| **OrganoID** (Matthews 2022) | 2022 | CNN | Yes | Yes | No | NR | Broad | Broad |
| **Tellu** (Domènech-Moreno 2023) | 2023 | YOLO | Yes | No | Yes | 23 066 (o[3]) | Specific | Specific |
| **OrgaExtractor** (Park 2023) | 2023 | CNN | No | Yes | No | NR | Specific | Specific |
| **Deep-Orga** (Leng 2024) | 2024 | YOLO | Yes | No | No | 14,242 (o[3]) | Specific | Broad |
| **OrgaSegment** (Lefferts 2024) | 2024 | CNN | Yes | Yes | No | 15,515 | Specific | Specific |
| **OrganoidNet** (Ferreira 2025) | 2025 | Autre (StarDist) | Yes | Yes | Yes | NR | Specific | Specific |
| **Fiji + ilastik** (Ostrop 2021) | 2021 | Autre (ilastik) | Yes | Yes | No | NR | Specific | Specific |
| **deepOrganoid** (Powell 2022) | 2022 | CNN | No | No | Yes | NR | Specific | specific |
| **OUR STUDY** | 2025 | YOLO / SAM | Yes | Yes | Yes | 573 | Broad | Broad |

**e**

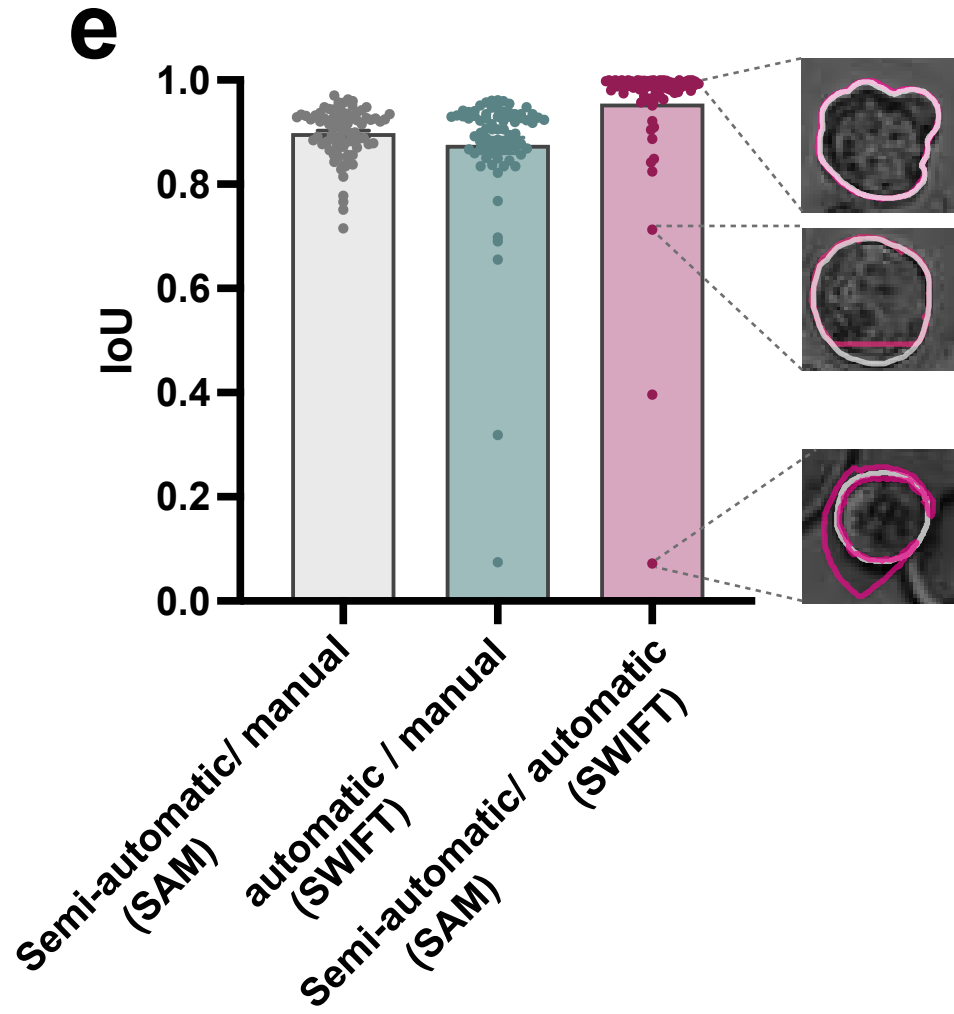

# SUPPLEMENTARY FIGURE 2

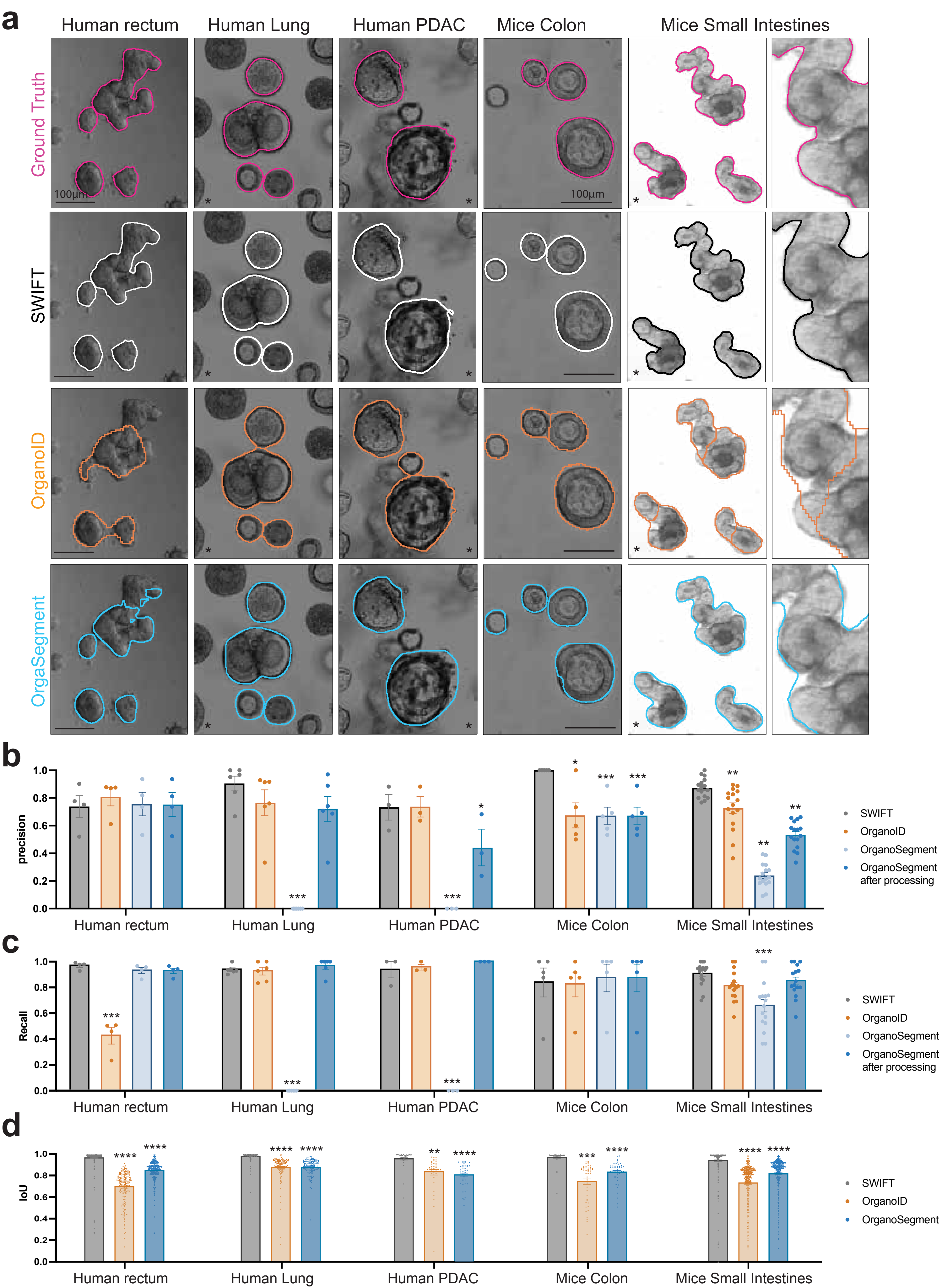

# SUPPLEMENTARY FIGURE 3

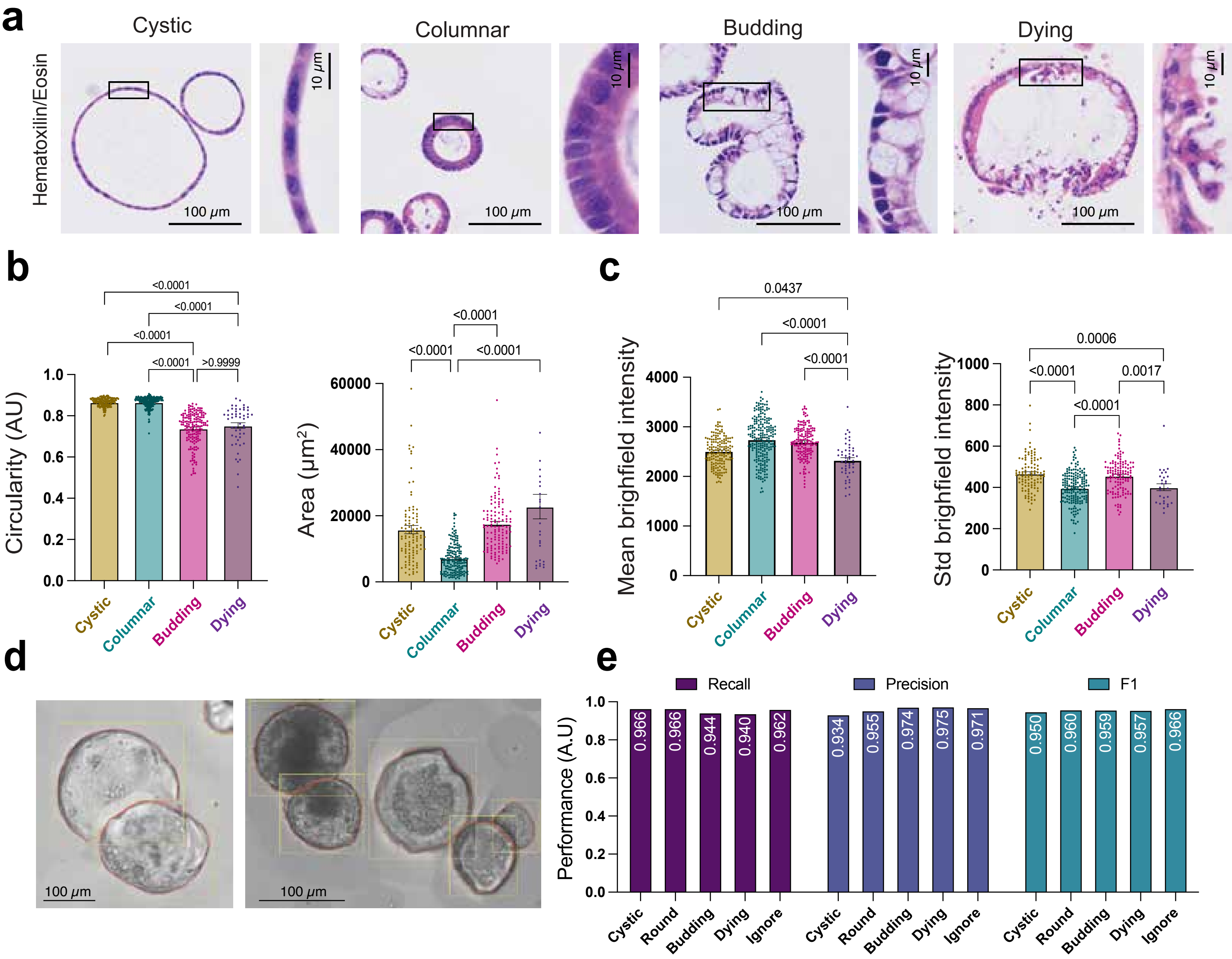

# SUPPLEMENTARY FIGURE 4

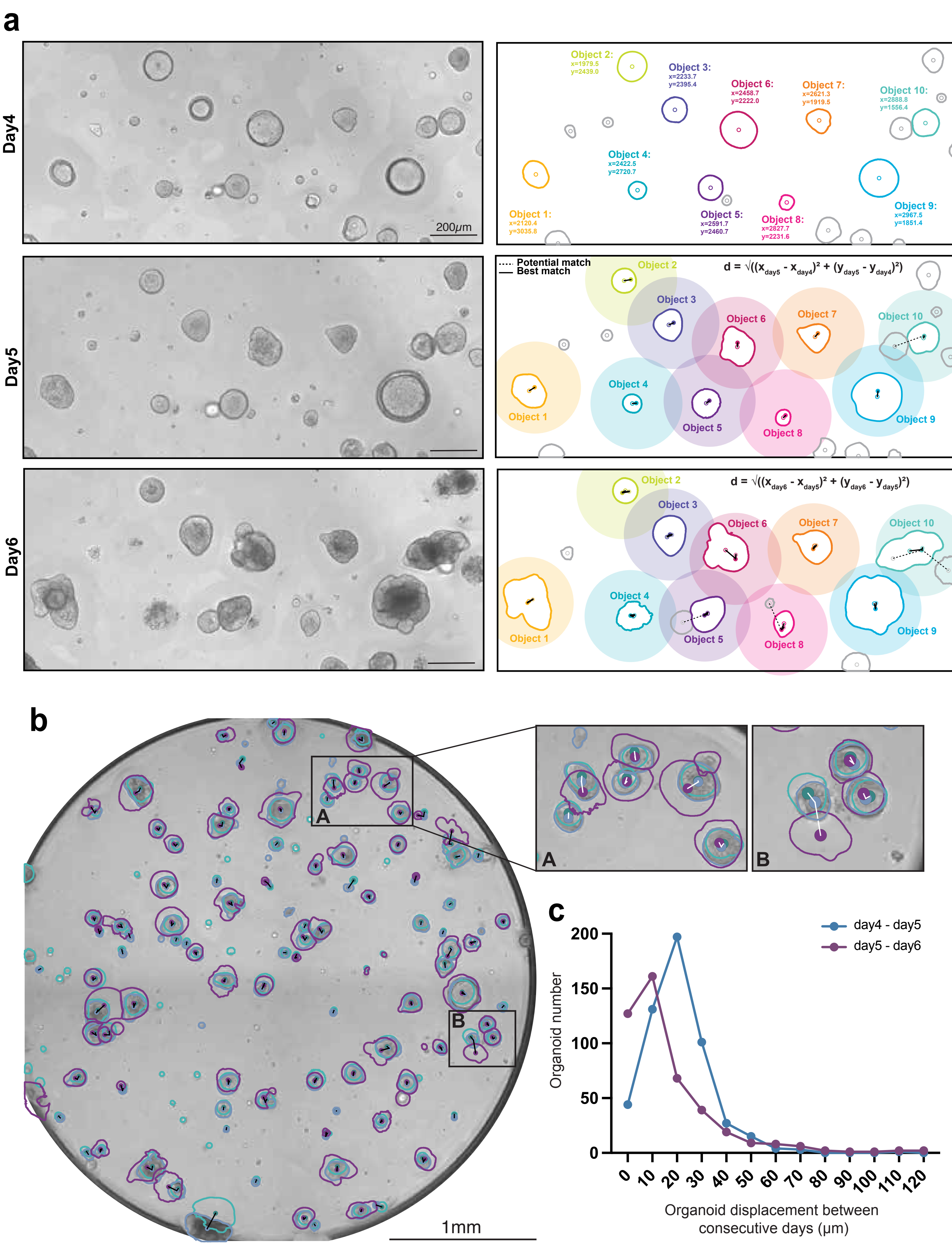

## SUPPLEMENTARY FIGURE 5

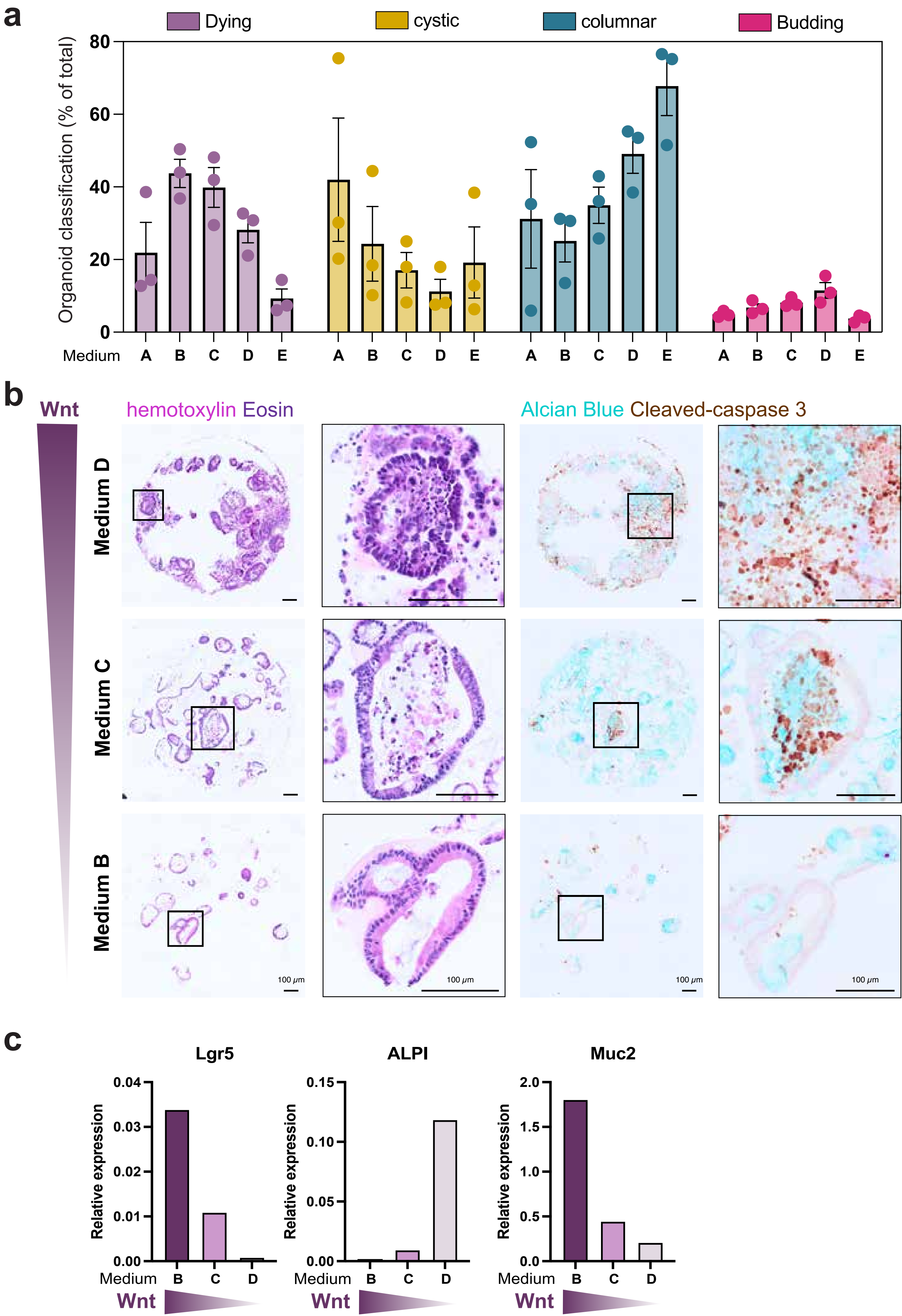

Supplement: Supplementary file 2 [file 42003_2026_10768_MOESM2_ESM.pdf]
