# Supplementary material for: A Single-organoid Workflow for quantitative Imaging classiFication and Tracking (SWIFT)
Source: Commun Biol. 2026 Sep 1;9:1149. doi: 10.1038/s42003-026-10768-x (PMC13534601; doi:10.1038/s42003-026-10768-x)
Supplement: Supplementary file 3 [file 42003_2026_10768_MOESM3_ESM.pdf]

Description of Additional Supplementary Files

File name : **Supplementary Video 1 |**
Description :

Supplementary Video 1 | Automated detection and segmentation of colonic organoids over a 67.5 h brightfield timelapse. Colonic organoids were grown in Medium C for 2 days and subsequently imaged for the following 67.5 h every 2.5 h in brightfield. Each timepoint was processed using the SWIFT pipeline, combining YOLOv8 for organoid detection and Segment Anything Model (SAM) for organoid segmentation. The resulting masks illustrate continuous detection and segmentation of individual organoids throughout the time course.

File name : **Supplementary Video 2 |**
Description :

Supplementary Video 2 | Automated classification and morphological state transitions of organoids over time. Using segmentation masks from Supplementary Video 1, each organoid was classified into the five morphological classes*: Cystic, Columnar, Budding*, *Dying*, or *Ignore*. Temporal evolution and transitions between classes were tracked and visualized, highlighting dynamic morphological changes over time (day 2 to day 5).

File name : **Supplementary Data 1 |**
Description : Source data
