# Supplementary material for: A Single-organoid Workflow for quantitative Imaging classiFication and Tracking (SWIFT)
Source: Commun Biol. 2026 Sep 1;9:1149. doi: 10.1038/s42003-026-10768-x (PMC13534601; doi:10.1038/s42003-026-10768-x)
Supplement: Supplementary file 4 [file 42003_2026_10768_MOESM7_ESM.pdf]

nature portfolio

Corresponding author(s): van der Goot, Gisou
Brandenberg, Nathalie

Last updated by author(s): 2026/04/24 by Brandenberg, Nathalie

Reporting Summary

Nature Portfolio wishes to improve the reproducibility of the work that we publish. This form provides structure for consistency and transparency in reporting. For further information on Nature Portfolio policies, see our Editorial Policies and the Editorial Policy Checklist.

Statistics

For all statistical analyses, confirm that the following items are present in the figure legend, table legend, main text, or Methods section.

n/a	Confirmed	
☐	☒	The exact sample size (*n*) for each experimental group/condition, given as a discrete number and unit of measurement
☐	☒	A statement on whether measurements were taken from distinct samples or whether the same sample was measured repeatedly
☐	☒	The statistical test(s) used AND whether they are one- or two-sided<br>*Only common tests should be described solely by name; describe more complex techniques in the Methods section.*
☐	☒	A description of all covariates tested
☐	☒	A description of any assumptions or corrections, such as tests of normality and adjustment for multiple comparisons
☐	☒	A full description of the statistical parameters including central tendency (e.g. means) or other basic estimates (e.g. regression coefficient) AND variation (e.g. standard deviation) or associated estimates of uncertainty (e.g. confidence intervals)
☐	☒	For null hypothesis testing, the test statistic (e.g. F, t, r) with confidence intervals, effect sizes, degrees of freedom and P value noted<br>*Give P values as exact values whenever suitable.*
☒	☐	For Bayesian analysis, information on the choice of priors and Markov chain Monte Carlo settings
☒	☐	For hierarchical and complex designs, identification of the appropriate level for tests and full reporting of outcomes
☐	☒	Estimates of effect sizes (e.g. Cohen's d, Pearson's r), indicating how they were calculated

Our web collection on statistics for biologists contains articles on many of the points above.

Software and code

Policy information about availability of computer code

Data collection

- Microscopy images were acquired:
on an inverted microscope (Nikon Ti2) equipped with transmitted light (DIA LED) and a 10× objective (Plan Apo λD 10×/0.45 NA, OFN25, DIC N1; objective code MRD70170) or specifically for Supplementary Figure 2a on an Olympus VS200 whole slide scanner using OlyVIA software and ×40 air objective.
External brightfield datasets were collected from Lefferts, J. W. et al. OrgaSegment: deep-learning based organoid segmentation to quantify CFTR dependent fluid secretion. Commun. Biol. 7, 319 (2024) and Matthews, J. M. et al. OrganoID: A versatile deep learning platform for tracking and analysis of single-organoid dynamics. PLoS Comput. Biol. 18, e1010584 (2022).
- Microscopy images were processed using NGFF Converter, the institutional OMERO server (EPFL, omero-server.epfl.ch), YOLOv8s network (Ultralytics v8.3.156), QuPath (v0.5.1) and the institutional workstation BIOP Desktop 21 (EPFL BioImaging & Optics Platform).
- Statistical data was retrieved using GraphPad Prism v10.2.2 (GraphPad Software).
- Analysis was performed and Sankey diagrams were generated in R

Data analysis

= CODE DESCRIPTION =
This modular workflow performs automated single-organoid detection, segmentation, classification and tracking from brightfield z-stack images. It combines Extended Depth-of-Field (EDF) projection using Fiji, YOLO-based object detection, SAM-based segmentation, and phenotype classification in QuPath.

== WORKFLOW STEPS ==

1. EXTENDED DEPTH-OF-FIELD PROJECTION (EDF) IN FIJI

Start Fiji and load raw brightfield z-stack images (.ND2 or .OME-TIFF).
Download and open the script `0-Fiji-EDF.groovy` in the Fiji Script Editor (drag & drop onto the main window). Press Run and specify EDF parameters including sigma (default σ = 50). The script applies a variance-based Extended Depth-of-Field projection (CLIJ2 GPU-accelerated) and exports pyramidal OME-TIFF images for analysis.

2. YOLO DETECTION & SAM SEGMENTATION IN QUPath
Start QuPath (v0.5.1) with the SAM extension enabled.
Create a new project and import EDF images generated in Fiji.
Download and open the script `1-QP-Project_YOLO-SAM.groovy` in the QuPath Script Editor.
Set the YOLO model filename through the variable `MODEL_NAME` (model stored in `project/models/` folder). Press Run to:
- run YOLO inference for bounding-box detection
- automatically segment each detected organoid using the Segment Anything Model (SAM) via the qupath-extension-sam plugin.

Resulting polygons are stored as objects within each image.

3. ORGANOID CLASSIFICATION IN QUPath
Manually annotate organoids into the categories to be classified.
Train the object classifier using `Analyze → Train object classifier`.
Convert annotations into detections using `2-QP-Convert_Annotations_to_Detections.groovy`.
Apply the classifier using `Analyze → Apply object classifier`.
Predicted classes include: Cystic, Columnar, Budding, Dying, Ignore.

== EXPECTED OUTPUT ==
Input EDF images → YOLO bounding boxes → SAM segmentation → Class-labeled organoid objects
Masks are saved as polygon detections and used for downstream quantification and tracking.

== OPTIONAL: YOLO MODEL FINE-TUNING ==
To fine-tune a YOLOv8 model:
1. Create a QuPath project with EDF images and manually annotated organoids.
2. Export training annotations using script: `1-QP-Export_training_annotation_to_train_YOLO.groovy`
3. Fine-tune the model using Ultralytics YOLO command such as: `yolo detect train data="path/data.yaml" model=yolov8s.pt epochs=70 imgsz=1024 augment=true`

== DEPENDENCIES ==
This workflow was tested and validated using the docker image "biop-desktop" version 0.2.3 (https://hub.docker.com/r/biop/biop-desktop), which contains all the necessary software of the workflow, including Fiji, QuPath (and its extensions), Yolo, SAM.

Data can be stored locally or on an OMERO server.

== SOFTWARE LIST ==

Software	Version	Notes
Fiji	stable release	CLIJ, CLIJx, CLIJ2, PTBIOP
QuPath	v0.5.1	with qupath-extension-sam
Ultralytics YOLOv8	v8.3.156	custom fine-tuning supported
samapi	0.6.1	object segmentation
R	v4.3.1	trajectory analysis
GraphPad Prism	v10.2.2	statistical analysis

== AVAILABILITY ==
All scripts used in this workflow will be deposited on GitHub upon publication.
GitHub: https://github.com/upvdg/swift-organoids

== AUTHOR INFORMATION ==
Workflow design & code: Lucie Bracq, Nathalie Brandenberg & Romain Guiet, EPFL BIOP & VDG

== COPYRIGHT ==
© EPFL BioImaging & Optics Platform (BIOP) & Global Health Institute (VDG), 2025. All rights reserved.

For manuscripts utilizing custom algorithms or software that are central to the research but not yet described in published literature, software must be made available to editors and reviewers. We strongly encourage code deposition in a community repository (e.g. GitHub). See the Nature Portfolio guidelines for submitting code & software for further information.

Data

Policy information about availability of data

All manuscripts must include a data availability statement. This statement should provide the following information, where applicable:

- Accession codes, unique identifiers, or web links for publicly available datasets
- A description of any restrictions on data availability
- For clinical datasets or third party data, please ensure that the statement adheres to our policy

All Raw data are provided has a Source data file. All Code details are provided. All data supporting the findings of this study are available within the paper, the Supplementary Information and Supplementary Data Source files.

Research involving human participants, their data, or biological material

Policy information about studies with human participants or human data. See also policy information about sex, gender (identity/presentation), and sexual orientation and race, ethnicity and racism.

Reporting on sex and gender	N/A
Reporting on race, ethnicity, or other socially relevant groupings	N/A
Population characteristics	N/A
Recruitment	N/A
Ethics oversight	N/A

Note that full information on the approval of the study protocol must also be provided in the manuscript.

Field-specific reporting

Please select the one below that is the best fit for your research. If you are not sure, read the appropriate sections before making your selection.

☒ Life sciences ☐ Behavioural & social sciences ☐ Ecological, evolutionary & environmental sciences

For a reference copy of the document with all sections, see nature.com/documents/nr-reporting-summary-flat.pdf

Life sciences study design

All studies must disclose on these points even when the disclosure is negative.

Sample size	All sample sizes and correspondent statistical comparisons are indicated in figure legends or methods sections.
Data exclusions	no data was excluded, and all experiment results are detailed
Replication	For all experiments we chose to analyses three or more biological replicates from two independent assays.
Randomization	non applicable to this study because human bias were not present in the presented measurements
Blinding	The image analysis was done automatedly

Reporting for specific materials, systems and methods

We require information from authors about some types of materials, experimental systems and methods used in many studies. Here, indicate whether each material, system or method listed is relevant to your study. If you are not sure if a list item applies to your research, read the appropriate section before selecting a response.

Materials & experimental systems

n/a	Involved in the study	
☐	☒	Antibodies
☒	☐	Eukaryotic cell lines
☒	☐	Palaeontology and archaeology
☐	☒	Animals and other organisms
☒	☐	Clinical data
☒	☐	Dual use research of concern
☒	☐	Plants

Methods

n/a	Involved in the study	
☒	☐	ChIP-seq
☒	☐	Flow cytometry
☒	☐	MRI-based neuroimaging

Antibodies

Antibodies used	Cleaved Caspase-3 Antibody ((Asp175) Cell Signaling: #9661, Citrate antigen retrieval, dilution 1:400. RRID: AB_2341188)
Validation	Cleaved Caspase-3 Antibody ((Asp175) Cell Signaling: #9661, Citrate antigen retrieval, dilution 1:400. RRID: AB_2341188). Independent validation by the NYU Lagone was performed for IHC. Detailed validation available on manufacturer's page: IHC analysis of paraffin embedded human tonsil, jurkat cells and mouse embryo.

Animals and other research organisms

Policy information about studies involving animals; ARRIVE guidelines recommended for reporting animal research, and Sex and Gender in Research

Laboratory animals	All laboratory animals were housed at 22°C +/- 1°C, 55% (+/-10%) humidity; with 12 h light (from 7 to 19 h) and 12 h dark (from 19 h to 7 h)<br>To generate colonic organoids, one 8 weeks-old C57BL/6j female was used.
Wild animals	No Wild animals were used in this study
Reporting on sex	Only one female was used
Field-collected samples	No field collected samples wher used in this study
Ethics oversight	Procedures were performed according to protocols approved by the Veterinary Authorities of the Canton Vaud and according to the Swiss Law (license VD3497, EPFL).

Note that full information on the approval of the study protocol must also be provided in the manuscript.

Plants

Seed stocks	N/A
Novel plant genotypes	N/A
Authentication	N/A
